# Mixing transcutaneous vagal nerve stimulation and galvanic cutaneous stimulation to decrease simulator adaptation syndrome

**DOI:** 10.3389/fpsyg.2024.1476021

**Published:** 2024-10-02

**Authors:** Germán Gálvez-García, Patricio Mena-Chamorro, Tomás Espinoza-Palavicino, Tatiana Romero-Arias, Mauricio Barramuño-Medina, Claudio Bascour-Sandoval

**Affiliations:** ^1^Departamento de Psicología, Universidad de La Frontera, Temuco, Chile; ^2^Departamento de Psicología Básica, Psicobiología y Metodología de las Ciencias del Comportamiento, Facultad de Psicología, Universidad de Salamanca, Campus Ciudad Jardín, Salamanca, Spain; ^3^Doctorado en Psicología, Universidad de La Frontera, Temuco, Chile; ^4^Facultad de Ciencias de la Salud, Universidad Europea de Canarias, La Orotava, Spain; ^5^Programa de Kinesiología, Facultad de Ciencias de la Salud, Universidad Autónoma de Chile, Temuco, Chile; ^6^Departamento de Ciencias de la Rehabilitación, Universidad de La Frontera, Temuco, Chile

**Keywords:** simulator adaptation syndrome, motion sickness, transcutaneous vagal nerve stimulation, galvanic cutaneous stimulation, neurostimulation

## Abstract

**Purpose:**

Simulator Adaptation Syndrome arises from a perceptual discordance between expected and actual motion, giving rise to symptoms such as nausea and disorientation. This research focused on determining the benefit of Transcutaneous Vagal Nerve Stimulation (tVNS) and Galvanic Cutaneous Stimulation (GCS), where both were applied in conjunction, as compared to their administration in isolation, to decrease Simulator Adaptation Syndrome (SAS).

**Method:**

A driving simulation study was proposed where SAS, body balance, and driving performance were measured. These measurements were taken during seven different stimulation scenarios with a baseline condition without stimulation compared against tVNS and GCS conditions.

**Results:**

The main result showed that the combination of tVNS and GCS reduced SAS and improved body balance and driving performance more successfully than their administration in isolation.

**Conclusion:**

Similar neuromodulation in the temporoparietal junction is proposed to mitigate SAS for GCS and tVNS (although additional explanations are discussed). Applying both techniques simultaneously is encouraged to decrease SAS in future interventions.

## 1 Introduction

Motion sickness (MS) arises from a perceptual discordance between expected and actual motion, giving rise to symptoms such as fatigue, cold sweats, pallor, dizziness, nausea, disorientation, vomiting, etc. (Keshavarz and Golding, 2022). In contrast, Simulator Adaptation Syndrome (SAS) is a variant of MS encountered within virtual simulators. Although the reported occurrence rates and severity level can vary significantly, it is important to note that premature termination rates during virtual reality sessions due to SAS can reach as high as 60% (Saredakis et al., 2020). Different theories have emerged to elucidate the origins of MS and SAS, such as postural instability theory (Riccio and Stoffregen, 1991) or the sensory conflict theory (Reason and Brand, 1975), where the etiology of SAS is described in terms of a conflict between the motion imposed by the simulator and the body’s natural balance (postural instability theory), or because a discrepancy between the individual’s prior experiences and the simulator’s visual, vestibular, or somatosensory feedback (sensory conflict theory).

Different studies have described countermeasures to reduce MS and SAS symptoms (see Keshavarz and Golding, 2022 for a current review of MS countermeasures) such as biofeedback and cognitive methods (Bos, 2015; Yen Pik Sang et al., 2003) or drugs (Lucot, 1998). Recently, using two countermeasures conjointly to reduce MS and/or SAS has proven highly effective. For example, Bos (2015) found that the use of head vibration and mental distraction reduced MS symptoms by 39% (although the research was not specific to SAS). In addition, it was found that vibration in isolation only reduced MS symptoms by 25% and mental distraction by 19%. Gálvez-García et al. (2015) studied the combination of Galvanic cutaneous stimulation (GCS) and auditory stimulation for SAS, finding an additive effect between both techniques with a 73% reduction of symptoms about the baseline condition, compared to lower effectiveness when both techniques were applied separately (50% for GCS and 48% for auditory stimulation). The authors concluded that both techniques additively improved body balance (i.e., measured through head movements) with a direct correlation with SAS symptoms. Moreover, the driving performance variables were not impaired, which is crucial to recommend these techniques to prevent SAS. Similarly, Gálvez-García et al. (2020a,b) studied the combination of GCS and tactile stimulation for SAS. They concluded that both techniques simultaneously decreased SAS more effectively (78%) than individual administration (i.e., 49 and 48% for GCS and tactile stimulation). The authors stated that GCS and tactile stimulation were additive to reducing SAS symptoms but did not find an additive effect on body balance. Whereas GCS improved body balance, tactile stimulation did not, concluding that the reduction in SAS for this technique was due to the distraction from its symptoms.

The above paragraph supports the notion of the advantage of using two countermeasures to reduce SAS. Moreover, the effectiveness of GCS in mitigating SAS has been widely tested. However, recent research (Espinoza-Palavicino et al., 2023) stated that Transcutaneous Vagal Nerve Stimulation (tVNS) showed better results at decreasing SAS than GCS (along with a better improvement in body control and driving performance variables).

The features of these techniques are outlined below. On the one hand, GCS stimulates (below the motor threshold) the cutaneous nerve fibers of the sternocleidomastoids by placing the electrodes 3–4 cm below the mastoid process. This area contains a high concentration of sensitive subcutaneous fibers (Lazorthes, 1981) that target the temporoparietal junction (Pérennou et al., 2001). The temporoparietal junction is involved in the processing of vestibular and visual information (Donaldson et al., 2015; Ventre-Dominey, 2014). This area is comparable to the parietal-insular-vestibular area identified in monkeys (Grüsser et al., 1990; Sugiuchi et al., 2005), which contains neurons that respond to somesthetic stimulation, particularly from the neck, in addition to visual and vestibular input. Thus, GCS would target these neurons at the temporoparietal junction, providing the central nervous system with information about the head and trunk position in space (Grüsser et al., 1990). In this line, GCS has been shown to improve balance in patients with neglect (Guariglia et al., 2000) and in fixed simulators where vestibular information is absent (e.g., Gálvez-García et al., 2020a,b). In addition, the impact of anodal transcranial direct current stimulation on the temporoparietal junction in virtual reality sickness has been directly tested (Takeuchi et al., 2018), where improvements in body balance and sickness were found. On the other hand, tVNS is an electrical stimulation technique that enables the direct stimulation of the vagus nerve via the acoustic meatus of the outer ear (Farmer et al., 2021). This ultimately results in the stimulation of the nucleus tractus solitarus and locus coeruleus, which leads to a significant release of gamma-aminobutyric acid (GABA) and norepinephrine (Warren et al., 2018). An increase in the levels of these neurotransmitters has been observed to result in enhanced cortical activity in several different structures, including the cerebellum and frontal lobes (Badran et al., 2018). In contrast to GCS, no direct relationship has been demonstrated between tVNS and temporoparietal junction activation in improving balance. Without ruling out this relationship (see, e.g., Fang et al., 2016, where tVNS modulates temporoparietal function), other possible mechanisms may influence the improvement of body balance by tVNS, such as activation in the nucleus tractus solitarus and locus coeruleus, which transmit afferent sensory information through connections with the thalamus, orbitofrontal cortex and medulla (George et al., 2000, Sigurdsson et al., 2021). In this vein, various studies have found that tVNS improves body balance (i.e., gait parameters) in people with Parkinson’s disease (Marano et al., 2022; Zhang et al., 2023), which is consistent with Espinoza Palavicino’s research finding improved body balance (along with reduced SAS symptomatology). Furthermore, tVNS can enhance concentration and cognitive flexibility (Borges et al., 2020; Colzato et al., 2018). This may mitigate the symptoms of anxiety and stress associated with SAS by focusing the participant’s attention on the task at hand.

In this context, we want to determine the effectiveness of tVNS and GCS in conjunction by decreasing SAS, having regard to the following as a base: (a) the effectiveness demonstrated by tVNS individually (e.g., Espinoza-Palavicino et al., 2023), and (b) the effectiveness of GCS individually (e.g., Gálvez-García et al., 2015) and in conjunction with other techniques (e.g., Gálvez-García et al., 2020a,b). Furthermore, body balance will be measured in both GCS and tVNS conditions through head balance. Finally, driving performance variables will be measured to test the impact of the combination of GCS and tVNS on driving behavior. Importantly, we will also measure the effectiveness of tVNS and GCS in isolation as a baseline to establish the hypothesized improvement of both techniques in conjunction. Indirectly, this will also provide further evidence for the previous results of Espinoza-Palavicino et al. (2023) about the effectiveness of GCS and especially tVNS in isolation. In short, we hypothesize that using both interventions in conjunction will positively affect the body balance, reducing SAS and increasing driving performance compared to administering both interventions in isolation.

## 2 Materials and methods

### 2.1 Participants

Forty-two healthy adults (mean age = 26.12 ± 3.95, 21 women) were recruited by non-probability intentional sampling. *A priori* power analysis with a medium effect size (f2 = 0.25), power = 0.95, *α* = 0.05 (F test family, repeated measures ANOVA; G*Power version 3.1; Faul et al., 2009) determined a sample size of 40 participants. However, two additional participants were recruited due to the counterbalancing of the experimental conditions to control for immediate carryover/adaptation effects. Thus, the order of the seven experimental conditions was counterbalanced following a Latin square design (Kim and Stein, 2009). The advantage of this method is the counterbalancing of immediate sequential effects in addition to ordinal positions. The design resulted in 42 counterbalanced sequences with the seven experimental conditions (which will be described later). All participants were right-handed, determined by the Spanish version of the Edinburgh Handedness Inventory (Albayay et al., 2019) and reported normal or corrected-to-normal vision. Before the experiment, participants completed the Motion Sickness Susceptibility Questionnaire (Golding, 1998). Participants with scores above 65 (75th percentile) or 0 were excluded from the study due to their heightened susceptibility or disinclination to experiencing SAS in the same order (7 participants). Based on previous research on SAS and GCS (e.g., Gálvez-García et al., 2020a,b; Reed-Jones et al., 2009) and tVNS (e.g., Beste et al., 2016; Colzato et al., 2018; Espinoza-Palavicino et al., 2023: Fischer et al., 2018), the following inclusion criteria were added: (a) normal state of health; (b) driving at least 3,000 km in the last 12 months; (c) no use of medication that impairs driving performance; (d) no history of neurological/psychiatric disorders or brain surgery (measured by the Mini International Neuropsychiatric Interview; Sheehan et al., 1998); (e) no migraine or epilepsy susceptibility, pregnancy, heart issues, ear alterations, or facial/brain metal implants. None of the participants had prior tVNS experience. Before each session, participants reported low state anxiety (i.e., scores between 20 and 37; Spielberger, 1970). This excludes it as an explanatory factor to the results. Withdrawal from the experiment was allowed without negative consequences at any time. After completing the experiment, participants were also informed about tVNS and sham stimulation types and the study’s purpose. All participants provided signed written informed consent. The *Universidad de La Frontera’s* Research Ethics Committee approved the study (N°078/23), conducted following the Declaration of Helsinki principles.

### 2.2 Apparatus and stimuli

#### 2.2.1 Driving simulator

**Figure 1:**
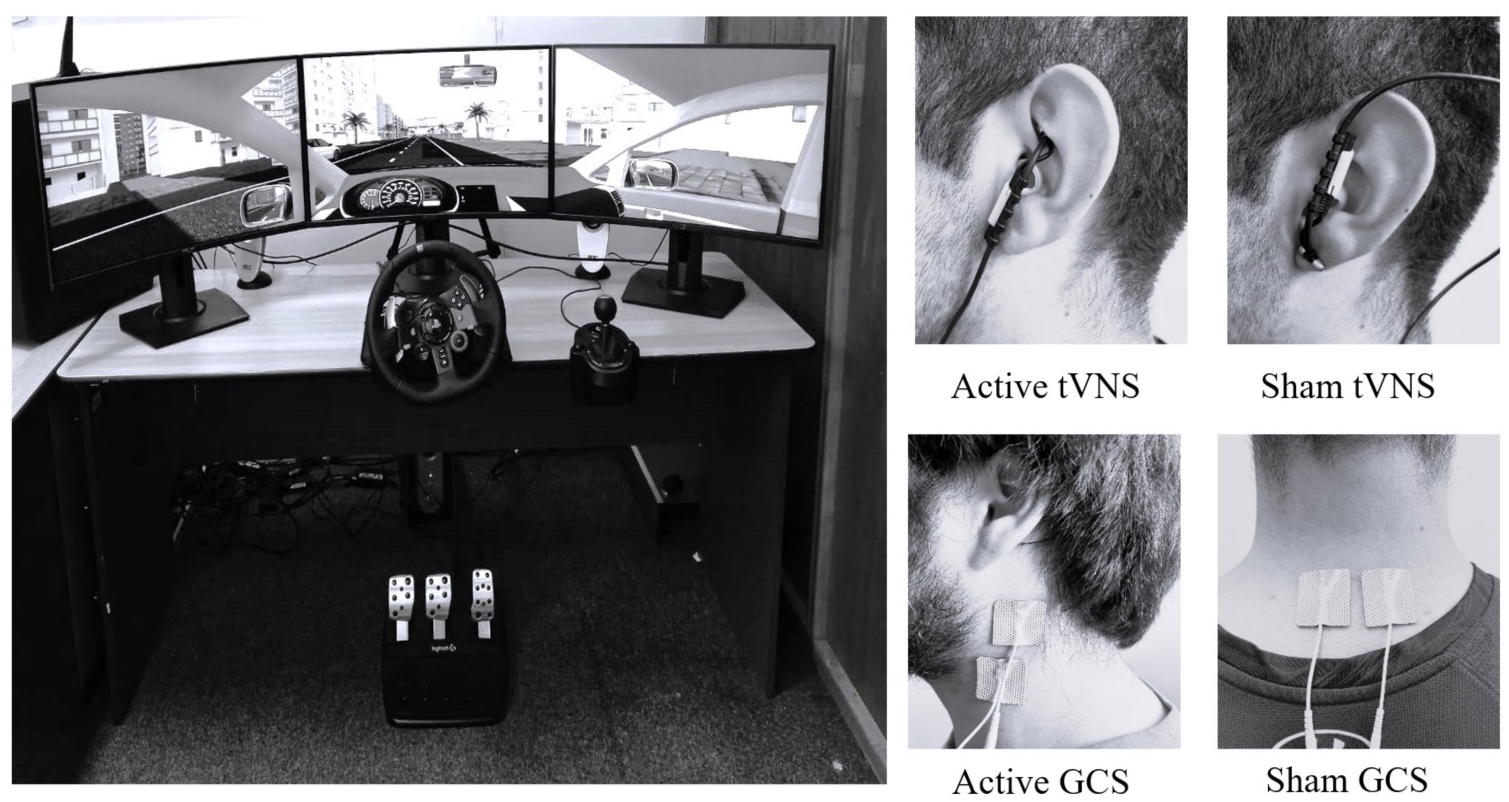
Driver simulator and electrode placement for each source of stimulation.

The driving scenario was similar to previous research (e.g., Espinoza-Palavicino et al., 2023). It had a 24.6 km flat route in an urban setting with 27 right and 27 left curves. Half of these curves were gradual (i.e., 70 m lead-in, a 140 m curve, and a 70 m lead-out), while the remaining 50% were sharp (i.e., 40 m lead-in and a 40 m curve). Straight sections ranging from 200 to 300 m were interspersed between the curves. The driving simulator (see Figure 1) consists of three 27-inch monitors, a steering wheel, pedals, and a gearbox. The central monitor had a screen viewing angle of 70° horizontally and 43° vertically. The seat height was aligned with the participant’s line of sight with the focal point on the apparent horizon line of the image displayed on that monitor. The other two screens flanked the central one. Carnetsoft software^1^ was used to create the driving simulation scenarios. The room temperature was controlled (mean temperature = 21 ± 1.2°C). Head movements were recorded using a high-speed camera (S-MOTION), and a Matlab algorithm measured the tip of the nose movements in the X-and Y-axes. Finally, the total scores from the Simulator Sickness Questionnaire (SSQ; Kennedy et al., 1993) were used to assess the SAS.

#### 2.2.2 Galvanic cutaneous stimulation

There were two active stimulation conditions regarding GCS. Active GCS and Active GCS + tVNS condition (see Figure 1 for accurate electrode placement for each source of stimulation). In addition, sham stimulations were measured for active control (sham GCS condition and sham GCS + tVNS condition, see Figure 1). These last conditions aim to control any placebo effects caused by electrical stimulation. Finally, a no-stimulation condition was included. In this section, we describe the features of GCS and how it is applied in the active and passive conditions previously stated (this information will be further extended in the following section on tVNS features).

Two electrodes (2.5 cm^2^) were placed symmetrically atop the sternocleidomastoid muscles for the active GCS conditions, situated approximately 3–4 cm beneath the mastoid process. The current output was delivered using the STMISOLA stimulus isolator (Biopac) determined by the participant’s detection threshold in line with previous protocols for active GCS conditions (e.g., Reed-Jones et al., 2008). This output was calibrated before the simulator task, ramping up by 0.05 mA as long as the participant made small head movements. Finally, the output was multiplied by two, which aligns with previous research that showed that this procedure provided a pleasant stimulation strength (Reed-Jones et al., 2008). The current range was between 1 and 1.7 mA. For sham GCS conditions, two GCS electrodes were positioned on each lateral side of the vertebra prominens (*C7*)*. This location was used because placing electrodes in other neck areas* (e.g., *trapezius*) possibly triggers the cranial portion of the spinal accessory nerve linked to the vagus nerve. This led to a different detection threshold procedure for GCS sham conditions than GCS active conditions because subcutaneous sensitive fibers were not stimulated (and no small head movements were performed to establish the detection threshold like GCS active conditions). Therefore, the same current output of active GCS conditions was chosen for sham GCS conditions by asking participants whether it produced a comfortable and suitable stimulation strength as in active GCS conditions. Only one participant reported an uncomfortable current output, so the stimulation was reduced by 0.1 mA (reporting with this reduction a comfortable and suitable stimulation strength). Thus, the current range was between 0.9 and 1.7 mA.

#### 2.2.3 Transcutaneous vagus nerve stimulation

There were two active stimulation conditions regarding tVNS. Active tVNS and Active GCS + tVNS condition (see Figure 1 for accurate electrode placement for each source of stimulation). In addition, sham stimulations were measured for active control (sham tVNS condition and sham GCS + tVNS condition, see Figure 1). These last conditions aim to control any placebo effects caused by electrical stimulation (Farmer et al., 2021). Finally, a no-stimulation condition was included (this condition was the same as described above in the section on GCS features). In this section, we describe the features of tVNS and how it is applied in the active and passive conditions previously stated.

The NEMOS^®^ tVNS neurostimulation device was applied in the left ear (i.e., through two titanium electrodes). The guidelines recommended by Farmer et al. (2021) for optimal vagus nerve stimulation were followed (i.e., a pulse width of 200–300 ms at 25 Hz with the stimulation delivered continuously in a series of 10-s increasing and decreasing trial series). The stimulation intensity was determined by the participant’s detection threshold (For more details, see the procedure used by Fischer et al., 2018). The stimulation was applied to the left cymba conchae in the active tVNS conditions and the left earlobe in the sham conditions. The left earlobe lacks vagal nerve fibers (Fallgatter et al., 2003), which leads to the inactivity of brain stem or cortex activation (Kraus et al., 2013). Stimulation was explicitly directed to the left ear, deliberately avoiding the right ear to prevent potential secondary cardiac effects (Nemeroff et al., 2006). The current output was delivered at a current ranging between 0.5 and 3.5 mA for the active tVNS condition and 0.7 and 3.8 mA for the sham tVNS condition.

Regarding the use of no stimulation condition, it should be noted that its inclusion is particularly crucial in studies investigating techniques that impact body balance and distraction in SAS. On the one hand, it serves as a neutral baseline where the stimulation of muscles conveying somatosensory information to the central nervous system regarding head or trunk position is intentionally avoided (Gálvez-García et al., 2020a,b). On the other hand, this neutral condition excludes distraction from SAS symptoms as a concurrent factor or explanation. Prior research has shown that individuals are less likely to experience SAS when their attention is focused on external events (Reason and Brand, 1975; Gálvez-García et al., 2017, 2020b). This is particularly relevant as it has been proved that sham GCS and tVNS conditions (i.e., the conditions used to control placebo caused by electrical stimulation) divert participants’ attention from SAS symptoms (Espinoza-Palavicino et al., 2023).

A similar setup was used to rule out the presence of the electrodes as an explanatory factor for the different experimental conditions. As an illustration, for the active tVNS condition, the electrode was correctly placed by adding the pair of GCS electrodes, which did not deliver any electrical current (i.e., counterbalancing active GCS and sham GCS electrode placements across participants). The same logic was followed when placing the electrodes for the rest of the stimulation conditions. Finally, for no stimulation condition, the electrodes (i.e., one for tVNS and two bilateral for GCS) without electricity current were placed, counterbalancing all possible active and passive GCS and tVNS placement combinations across participants (i.e., sham GCS + sham tVNS placements, sham GCS + active tVNS placements, active GCS + sham tVNS placements, and active GCS + active tVNS placements).

#### 2.2.4 Head sway

A high-speed digital camera (S-MOTION) positioned centrally above the screen captured head movements, specifically the nose (measured in pixels along the X-and Y-axes). An algorithm developed in MATLAB software processed the recorded data (The MathWorks, 2012).

#### 2.2.5 Simulator sickness questionnaire

The total scores of the SSQ (Kennedy et al., 1993) were measured to assess SAS at the end of each experimental condition. Participants rated 16 symptoms on a scale from 0 (“none”) to 3 (“severe”), with higher scores indicating higher SAS severity.

### 2.3 Procedure

The experiment had seven experimental conditions (i.e., no stimulation condition, sham GCS condition, sham tVNS condition, sham GCS + tVNS condition, active GCS condition, active tVNS condition, active GCS + tVNS condition). The corresponding stimulation was administered during the five-minute familiarization session before and throughout each experimental condition. They were instructed to drive at a minimum speed of 80 km/h on the right side of the road and not to decrease their speed below 70 km/h during turns. Lead-ins and curves with speeds under 80 km/h and 70 km/h, respectively, were not factored into the analyses (35 lead-ins and 11 curves across all participants). These speed constraints were fundamental to homogenizing the task across participants and avoiding the differences in driving between them. In other words, different speeds (especially on curves) could lead to confusion or interference with the sickness ratings. The seven experimental conditions were performed with four-day intervals between each one to control sickness accumulation (Dużmańska et al., 2018).

### 2.4 Statistical analyses

The seven stimulation conditions represented the independent variables. Dependent variables included the total scores derived from SSQ. Additionally, head sway was provided as an objective measure of SAS (i.e., quantified as the standard deviation of head movements measured in pixels along the X-and Y-axes during circuit curves; Gálvez-García et al., 2015). As previously noted, head sway is positively associated with SAS symptoms (i.e., increased head sway as a compensatory response to the absence of natural motion results in an increase in SAS symptoms). Finally, the average speed (km/h) and the steering wheel variability of the curves (*SD* of steering wheel position) were measured as dependent variables. We used RStudio (version 2023.12.0) to perform linear mixed-effects models (LME) for the dependent variables. The estimated LME models included the factor “condition” as a fixed effect and “participants” as a random effect (e.g., Gálvez-García et al., 2020b). The computed LME models were compared using the Akaike information criterion (AIC) (e.g., the model with the lowest AIC was considered the best fitting and adjusted model). The exponent of the difference between the AIC models was then estimated to factor in the likelihood and parsimony of a given model [AICRL = exp.(ΔAIC/2)] (e.g., Albayay et al., 2022; Espinoza-Palavicino et al., 2023). Additionally, we calculated the marginal and conditional *R*^2^ to address the variance explained by the fixed effect (*R*^2^_m_) and the combined fixed and random effects (*R*^2^_c_). Multiple comparisons were conducted using the Tukey method to adjust *p*-values. It should be clarified that *post hoc* comparisons are not simply paired samples t-tests. For these comparisons, the emmeans function was employed through Satterthwaite approximation. Thus, the degrees of freedom within linear mixed-effects models depend not only on the number of subjects but also on the entire structure of the model, which includes both fixed and random effects. Spearman’s rho correlation coefficients (rs) were calculated within each experimental condition to ascertain the correlation between the SSQ score, head sway in both the X and Y directions, and driving performance variables for each experimental scenario.

## 3 Results

### 3.1 SSQ scores

**Figure 2:**
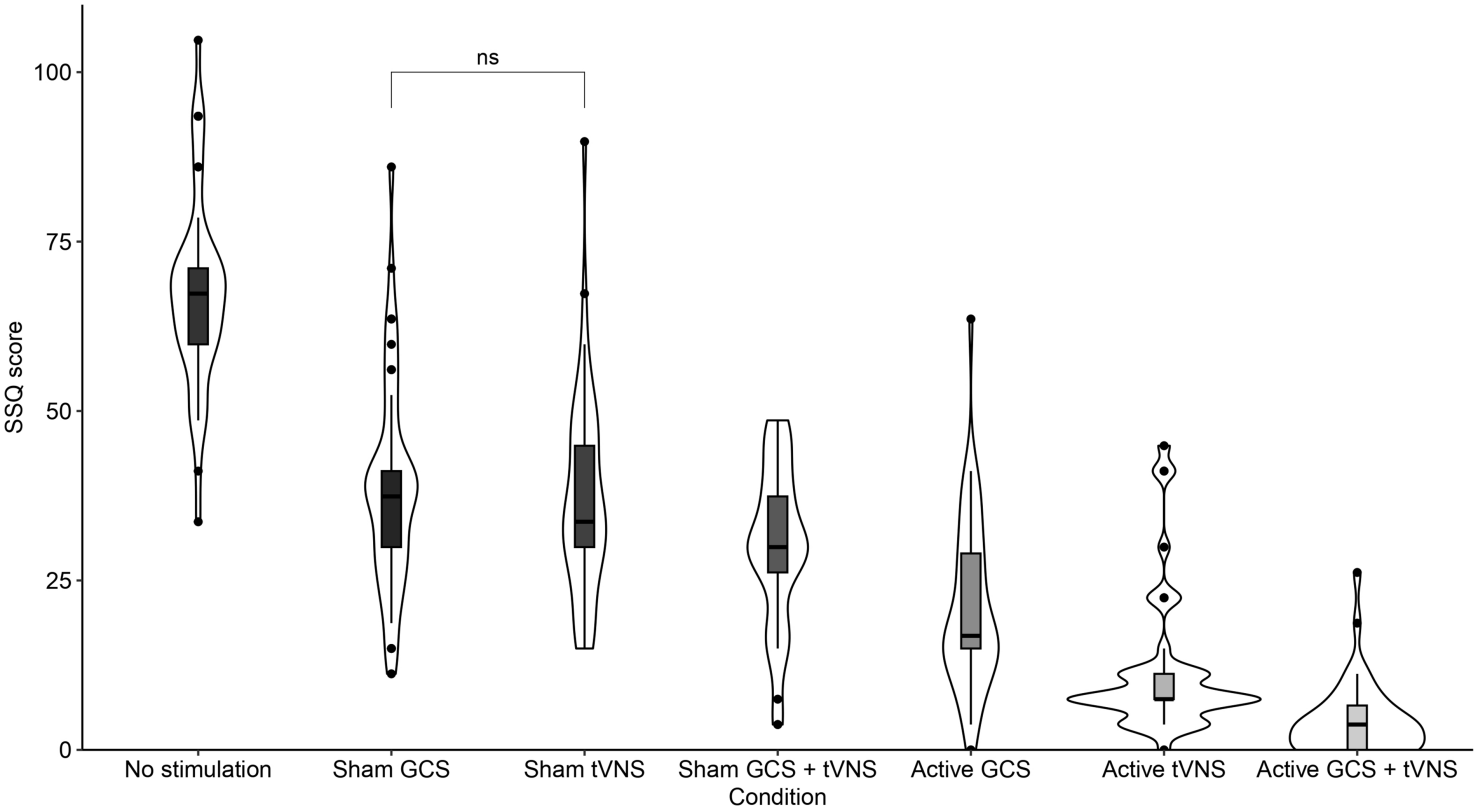
SSQ score per condition. No significant planned comparisons are highlighted.

Details of the descriptive statistics and LME model for SSQ scores are shown in Table 1. The main effect of the condition on the SSQ score was statistically significant (see Figure 2). Multiple comparisons showed lower SSQ scores in active GCS + tVNS condition as compared with the rest of conditions; no stimulation condition (*t*_(258)_ = 28.727, *p* < 0.001), sham GCS condition (*t*_(258)_ = 15.688, *p* < 0.001), sham tVNS condition (*t*_(258)_ = 14.995, *p* < 0.001), sham GCS + tVNS condition (*t*_(258)_ = 11.735, *p* < 0.001), active GCS condition (*t*_(258)_ = 7.661, *p* < 0.001), and finally active tVNS condition (*t*_(258)_ = 3.341, *p* = 0.016). SSQ scores were also significantly lower for active tVNS condition as compared to the rest of conditions; no stimulation condition (*t*_(258)_ = 25.386, *p* < 0.001), sham GCS condition (*t*_(258)_ = 12.347, *p* < 0.001), sham tVNS condition (*t*_(258)_ = 11.654, *p* < 0.001), sham GCS + tVNS condition (*t*_(258)_ = 8.394, *p* < 0.001), an finally active GCS condition (*t*_(258)_ = 4.319, *p* < 0.001). SSQ scores were also significantly lower for active GCS condition as compared to the rest of conditions; no stimulation condition (*t*_(258)_ = 21.067, *p* < 0.001), sham GCS condition (*t*_(258)_ = 8.027, *p* < 0.001), sham tVNS condition (*t*_(258)_ = 7.335, *p* < 0.001), and finally sham GCS + tVNS condition (*t*_(258)_ = 4.075, *p* = 0.001). In this vein, SSQ scores were also significantly lower for the sham GCS + tVNS condition as compared to the rest of the conditions; no stimulation condition (*t*_(258)_ = 16.992, *p* < 0.001), sham GCS condition (*t*_(258)_ = 3.953, *p* = 0.001) and finally sham tVNS condition (*t*_(258)_ = 3.260, *p* = 0.021). SSQ scores were significantly lower for the sham tVNS condition as compared to no stimulation condition (*t*_(258)_ = 13.732, *p* < 0.001), but there was no difference with the sham GCS condition (*t*_(258)_ = 0.693, *p* = 0.992). Lastly, the SSQ scores were significantly lower for the sham GCS condition as compared to the no stimulation condition (*t*_(258)_ = 13.039, *p* < 0.001). In short, the administration of GCS + tVNS was more effective at decreasing SAS symptoms, even though all other countermeasures also decreased SAS symptomatology.

**Table 1:** Results of the mixed-effects modeling.

Dependent variable	Independent variable	Mean ± SD	Likelihood ratio test	*p-*value	AIC_RL_	*R* ^2^_m_	*R* ^2^_c_
SSQ score	Condition		χ^2^(6) = 424.49	**<0.001**	<0.001	0.705	0.813
	No stimulation	67.05 ± 14.27					
	Sham GCS	38.56 ± 15.62					
	Sham tVNS	37.04 ± 15.14					
	Sham GCS + tVNS	29.92 ± 11.80					
	Active GCS	21.02 ± 12.53					
	Active tVNS	11.58 ± 10.44					
	Active GCS + tVNS	4.27 ± 5.39					
Head sway along the X-axis	Condition		χ^2^(6) = 268.70	**<0.001**	<0.001	0.555	0.661
	No stimulation	11.51 ± 5.00					
	Sham GCS	12.17 ± 4.20					
	Sham tVNS	12.47 ± 3.66					
	Sham GCS + tVNS	11.98 ± 2.69					
	Active GCS	7.59 ± 2.88					
	Active tVNS	4.56 ± 3.47					
	Active GCS + tVNS	2.15 ± 1.92					
Head sway along the Y-axis	Condition		χ^2^(6) = 266.09	**<0.001**	<0.001	0.531	0.671
	No stimulation	6.38 ± 2.06					
	Sham GCS	6.11 ± 2.13					
	Sham tVNS	6.32 ± 1.58					
	Sham GCS + tVNS	6.25 ± 1.73					
	Active GCS	4.38 ± 1.20					
	Active tVNS	3.49 ± 0.76					
	Active GCS + tVNS	2.16 ± 0.69					
Average speed (km/h)	Condition		χ^2^(6) = 403.81	**<0.001**	<0.001	0.701	0.794
	No stimulation	76.94 ± 2.69					
	Sham GCS	82.46 ± 3.75					
	Sham tVNS	82.53 ± 4.48					
	Sham GCS + tVNS	87.85 ± 5.26					
	Active GCS	91.55 ± 6.04					
	Active tVNS	95.34 ± 4.25					
	Active GCS + tVNS	99.40 ± 6.53					
Steering wheel variability	Condition		χ^2^(6) = 377.78	**<0.001**	<0.001	0.684	0.770
	No stimulation	22.72 ± 6.58					
	Sham GCS	31.70 ± 7.95					
	Sham tVNS	32.34 ± 6.14					
	Sham GCS + tVNS	37.39 ± 7.22					
	Active GCS	43.66 ± 8.20					
	Active tVNS	53.79 ± 12.57					
	Active GCS + tVNS	62.19 ± 10.63					

Bold *p* values denote statistical significance with α = 0.05.

### 3.2 Head sway

**Figure 3:**
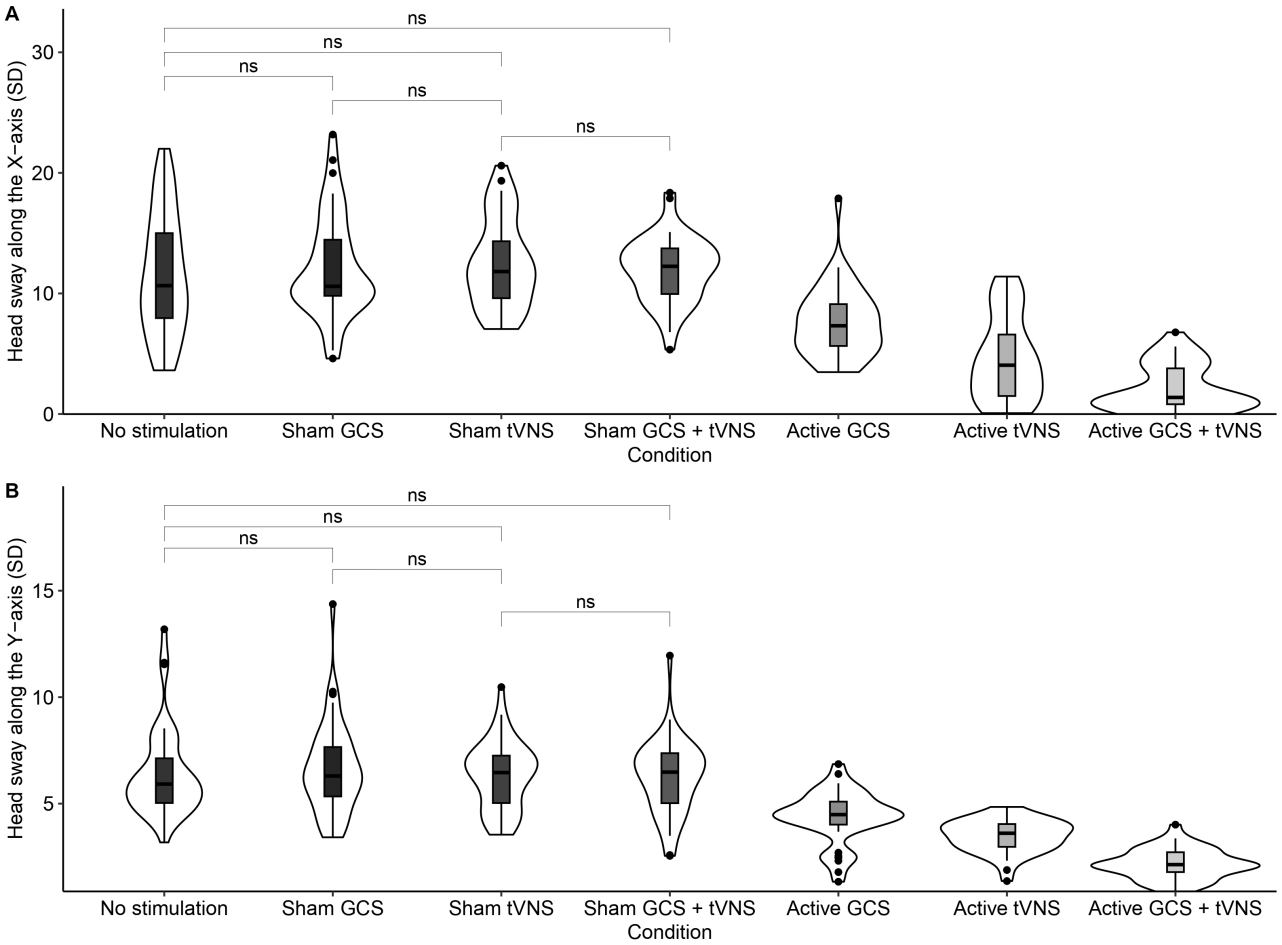
**(A)** Head sway along the X-axis, and **(B)** Head sway along the Y-axis per condition. No significant planned comparisons are highlighted.

Details of the descriptive statistics and LME models for head sway along the X-axis and Y-axis are shown in Table 1. The main effect of the condition on the head sway along the X-axis was statistically significant (see Figure 3A). Multiple comparisons showed lower head sway for active GCS + tVNS condition as compared to no stimulation condition (*t*_(258)_ = 13.934, *p* < 0.001), sham GCS condition (*t*_(258)_ = 14.911, *p* < 0.001), sham tVNS condition (*t*_(258)_ = 15.348, *p* < 0.001), sham GCS + tVNS condition (*t*_(258)_ = 14.618, *p* < 0.001), active GCS condition (*t*_(258)_ = 8.088, *p* < 0.001), and finally active tVNS condition (*t*_(258)_ = 3.588, *p* = 0.007). Lower head sway was also found for active tVNS condition as compared to no stimulation condition (*t*_(258)_ = 10.346, *p* < 0.001), sham GCS condition (*t*_(258)_ = 11.323, *p* < 0.001), sham tVNS condition (*t*_(258)_ = 11.760, *p* < 0.001), sham GCS + tVNS condition (*t*_(258)_ = 11.030, *p* < 0.001), and finally active GCS (*t*_(258)_ = 4.500, *p* < 0.001). Head sway was also significantly lower for active GCS condition as compared to no stimulation condition (*t*_(258)_ = 5.845, *p* < 0.001), sham GCS condition (*t*_(258)_ = 6.823, *p* < 0.001), sham tVNS condition (*t*_(258)_ = 7.260, *p* < 0.001), and finally sham GCS + tVNS condition (*t*_(258)_ = 6.530, *p* < 0.001). Instead, there was no significant difference in head sway between sham GCS + tVNS condition as compared to baseline and the rest of the sham conditions: no stimulation condition (*t*_(258)_ = −0.685, *p* = 0.993), sham GCS condition (*t*_(258)_ = 0.293, *p* = 0.998), and sham tVNS condition (*t*_(258)_ = 0.730, *p* < 0.990). There was also no significant difference in head sway between sham tVNS condition as compared to no stimulation condition (*t*_(258)_ = −1.414, *p* = 0.793) and sham GCS condition (*t*_(258)_ = −0.437, *p* = 0.995). Lastly, there was also no significant difference between the sham GCS condition and the no-stimulation condition (*t*_(258)_ = −0.978, *p* < 0.958).

The main effect of condition on the head sway along the Y-axis was also statistically significant (see Figure 3B), with lower head sway in the active GCS + tVNS condition as compared to the rest of conditions; no stimulation condition (*t*_(258)_ = 14.928, *p* < 0.001), sham GCS condition (*t*_(258)_ = 15.716, *p* < 0.001), sham tVNS condition (*t*_(258)_ = 14.682, *p* < 0.001), sham GCS + tVNS condition (*t*_(258)_ = 14.417, *p* < 0.001), active GCS condition (*t*_(258)_ = 7.842, *p* < 0.001), and finally active tVNS condition (*t*_(258)_ = 4.684, *p* < 0.001). Head sway was also significantly lower for active tVNS condition as compared to no stimulation condition (*t*_(258)_ = 10.244, *p* < 0.001), sham GCS condition (*t*_(258)_ = 11.033, *p* < 0.001), sham tVNS condition (*t*_(258)_ = 9.998, *p* < 0.001), sham GCS + tVNS condition (*t*_(258)_ = 9.733, *p* < 0.001), and finally active GCS condition (*t*_(258)_ = 3.158, *p* = 0.029). Head sway was also significantly lower for active GCS condition as compared to no stimulation condition (*t*_(258)_ = 7.086, *p* < 0.001), sham GCS condition (*t*_(258)_ = 7.875, *p* < 0.001), sham tVNS condition (*t*_(258)_ = 6.840, *p* < 0.001), and finally sham GCS + tVNS condition (*t*_(258)_ = 6.575, *p* < 0.001). In contrast, there was no significant difference in head sway between sham GCS + tVNS condition as compared to baseline and the rest of the sham conditions; no stimulation condition (*t*_(258)_ = 0.511, *p* = 0.998), sham GCS condition (*t*_(258)_ = 1.300, *p* = 0.851) and sham tVNS condition (*t*_(258)_ = 0.265, *p* = 0.998). There was also no significant difference in head sway between the sham tVNS condition as compared to the no stimulation condition (*t*_(258)_ = 0.246, *p* = 0.998) and sham GCS condition (*t*_(258)_ = 1.035, *p* = 0.945). Finally, there was no significant difference between the sham GCS and no stimulation conditions (*t*_(258)_ = −0.789, *p* = 0.985). To sum up, active GCS + tVNS condition, active tVNS condition, and active GCS condition yielded lower head sway along the X-axis and the Y-axis, especially the former.

### 3.3 Driving performance variables

**Figure 4:**
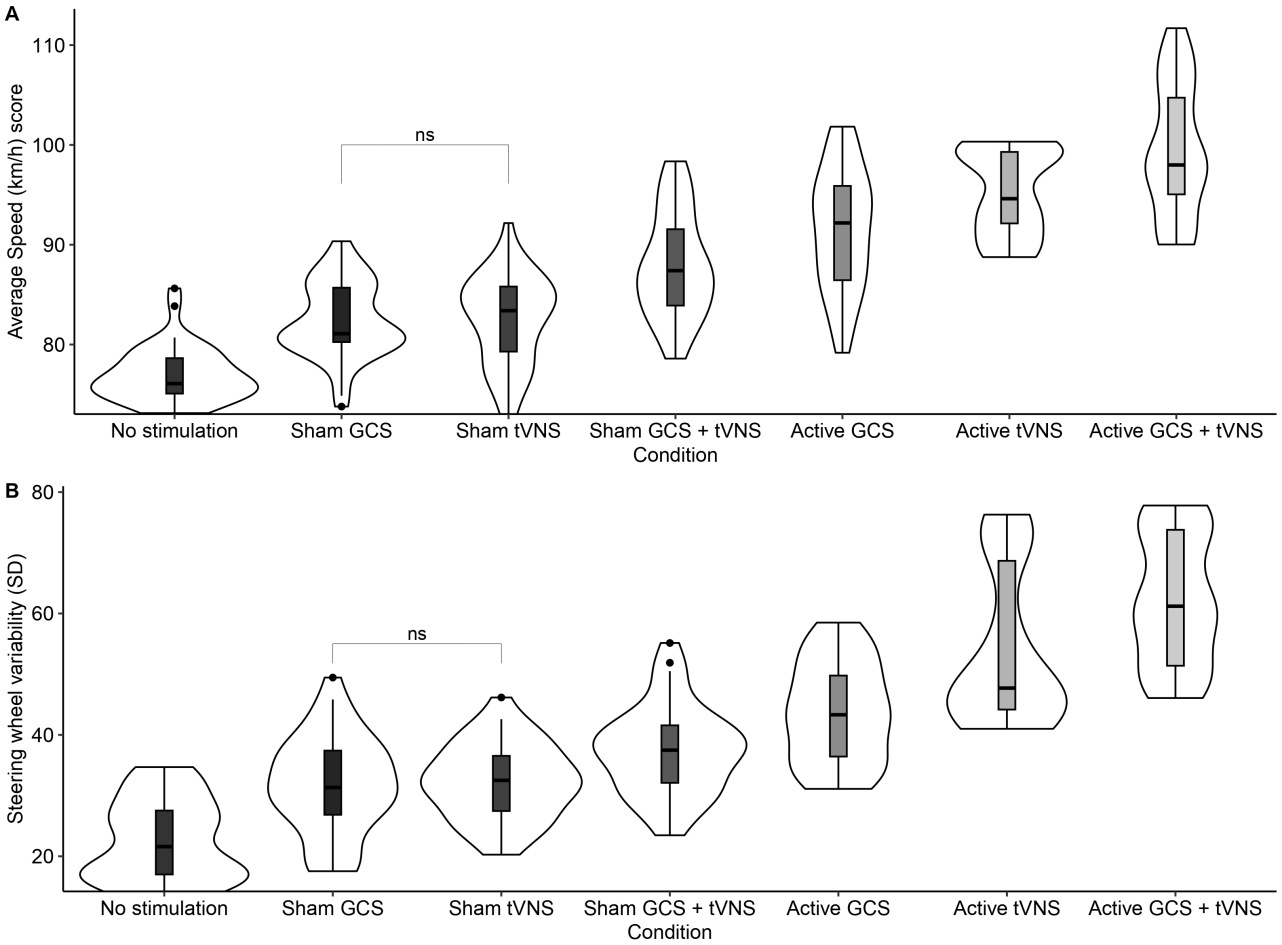
**(A)** Average speed, and **(B)** Steering wheel variability per condition. No significant planned comparisons are highlighted.

Details of the descriptive statistics and LME models for average speed and steering wheel variability are shown in Table 1. The main effect of the condition on the average speed was statistically significant (see Figure 4A). Multiple comparisons showed higher average speed in active GCS + tVNS condition than in no stimulation condition (*t*_(258)_ = −25.431, *p* < 0.001), sham GCS condition (*t*_(258)_ = −19.178, *p* < 0.001), sham tVNS condition (*t*_(258)_ = −19.098, *p* < 0.001), sham GCS + tVNS condition (*t*_(258)_ = −13.080, *p* < 0.001), active GCS condition (*t*_(258)_ = −8.890, *p* < 0.001), and finally active tVNS condition (*t*_(258)_ = −4.594, *p* < 0.001). Average speed was also significantly higher for active tVNS condition as compared to no stimulation condition (*t*_(258)_ = −20.837, *p* < 0.001), sham GCS condition (*t*_(258)_ = −14.584, *p* < 0.001), sham tVNS condition (*t*_(258)_ = −14.505, *p* < 0.001), sham GCS + tVNS condition (*t*_(258)_ = −8.487, *p* < 0.001), and finally active CGS (*t*_(258)_ = −4.296, *p* < 0.001). Average speed was also significantly lower for active GCS condition as compared to no stimulation condition (*t*_(258)_ = −16.541, *p* < 0.001), sham GCS (*t*_(258)_ = −10.288, *p* < 0.001), sham tVNS (*t*_(258)_ = −10.208, *p* < 0.001), and finally sham GCS + tVNS (*t*_(258)_ = −4.190, *p* < 0.001). Average speed was also significantly higher for sham GCS + tVNS condition as compared to no stimulation condition (*t*_(258)_ = −12.350, *p* < 0.001), sham GCS condition (*t*_(258)_ = −6.098, *p* < 0.001) and finally sham tVNS condition (*t*_(258)_ = −6.018, *p* < 0.001). Average speed was significantly higher for the sham tVNS condition as compared to no stimulation condition (*t*_(258)_ = −6.332, *p* < 0.001), but there was no difference with the sham GCS condition (*t*_(258)_ = −0.080, *p* = 0.995). Lastly, the average speed was significantly higher for the sham GCS condition as compared to the no-stimulation condition (*t*_(258)_ = −6.253, *p* < 0.001).

The main effect of condition on the steering wheel variability was also statistically significant (see Figure 4B), with higher variability in the active GCS + tVNS condition as compared to no stimulation condition (*t*_(258)_ = −24.270, *p* < 0.001), sham GCS condition (*t*_(258)_ = −18.751, *p* < 0.001), sham tVNS condition (*t*_(258)_ = −18.355, *p* < 0.001), sham GCS + tVNS condition (*t*_(258)_ = −15.248, *p* < 0.001), active GCS (*t*_(258)_ = −11.394, *p* < 0.001), and finally active tVNS condition (*t*_(258)_ = −5.162, *p* < 0.001). Steering wheel variability was also significantly higher for active tVNS condition as compared to no stimulation condition (*t*_(258)_ = −19.108, *p* < 0.001), sham GCS condition (*t*_(258)_ = −13.589, *p* < 0.001), sham tVNS (*t*_(258)_ = −13.194, *p* < 0.001), sham GCS + tVNS condition (*t*_(258)_ = −10.086, *p* < 0.001), and active GCS condition (*t*_(258)_ = −6.232, *p* < 0.001). Steering wheel variability was also significantly higher for active GCS condition as compared to no stimulation condition (*t*_(258)_ = −12.876, *p* < 0.001), sham GCS condition (*t*_(258)_ = −7.357, *p* < 0.001), sham tVNS condition (*t*_(258)_ = −6.961, *p* < 0.001), and finally sham GCS + tVNS condition (*t*_(258)_ = −3.854, *p* = 0.002). Steering wheel variability was also significantly higher for the sham GCS + tVNS condition as compared to baseline and the rest of the sham conditions; no stimulation condition (*t*_(258)_ = −9.022, *p* < 0.001), sham GCS condition (*t*_(258)_ = −3.503, *p* = 0.009) and finally sham tVNS condition (*t*_(258)_ = −3.108, *p* = 0.033). Steering wheel variability was significantly higher for the sham tVNS condition as compared to the no-stimulation condition (*t*_(258)_ = −5.915, *p* < 0.001). However, there was no difference with the sham GCS condition (*t*_(258)_ = 0.396, *p* = 0.997). Finally, steering wheel variability was significantly higher for sham GCS condition as compared to no stimulation condition (*t*_(258)_ = −5.519, *p* < 0.001). In brief, driving performance improved when active GCS + tVNS stimulation was administered, although all other countermeasures also improved speed and steering wheel variability.

### 3.4 Correlations

Details of the Spearman’s rho correlation coefficients are shown in Table 2. SSQ score and head sway were positively associated. For the X-axis, the association was large for no stimulation, sham GCS, sham tVNS, sham GCS + tVNS, active GCS, active tVNS, and active GCS + tVNS. For the Y-axis, the association was medium for active GCS + tVNS and large for no stimulation, sham GCS, sham tVNS, sham GCS + tVNS, active GCS, and active tVNS. On the other hand, SSQ scores and driving performance were negatively associated. For average speed, the association was large for no stimulation, sham GCS, sham tVNS, sham GCS + tVNS, active GCS, active tVNS, and active GCS + tVNS. SSQ score and Steering wheel variability were negatively associated as well. The association was large for no stimulation, sham GCS, sham tVNS, sham GCS + tVNS, active GCS, active tVNS, and active GCS + tVNS.

**Table 2:** Spearman’s rho correlation coefficients for the association between SSQ score, head sway along the X-and Y-axes, and driving performance variables per condition.

Condition	Head sway		Average speed	Steering wheel variability
	X-axis	Y-axis		
No stimulation	*r*_s_(42) = 0.67, *p* < 0.001	*r*_s_(42) = 0.70, *p* < 0.001	*r*_s_(42) = −0.70, *p <* 0.001	*r*_s_(42) = −0.82, *p* < 0.001
Sham GCS	*r*_s_(42) = 0.66, *p* < 0.001	*r*_s_(42) = 0.59, *p <* 0.001	*r*_s_(42) = −0.74, *p* < 0.001	*r*_s_(42) = −0.75, *p* < 0.001
Sham tVNS	*r*_s_(42) = 0.83, *p* < 0.001	*r*_s_(42) = 0.69, *p* < 0.001	*r*_s_(42) = −0.82, *p* < 0.001	*r*_s_(42) = −0.57, *p* < 0.001
Sham GCS + tVNS	*r*_s_(42) = 0.63, *p* < 0.001	*r*_s_(42) = 0.78, *p* < 0.001	*r*_s_(42) = −0.77, *p* < 0.001	*r*_s_(42) = −0.74, *p* < 0.001
Active GCS	*r*_s_(42) = 0.78, *p* < 0.001	*r*_s_(42) = 0.61, *p* < 0.001	*r*_s_(42) = −0.67, *p* < 0.001	*r*_s_(42) = −0.80, *p* < 0.001
Active tVNS	*r*_s_(42) = 0.83, *p* < 0.001	*r*_s_(42) = 0.69, *p* < 0.001	*r*_s_(42) = −0.82, *p* < 0.001	*r*_s_(42) = −0.96, *p* < 0.001
Active GCS + tVNS	*r*_s_(42) = 0.61, *p* = 0.001	*r*_s_(42) = 0.49, *p* = 0.001	*r*_s_(42) = −0.60, *p* < 0.001	*r*_s_(42) = −0.59, *p* < 0.001

## 4 Discussion

Espinoza-Palavicino et al. (2023) stated the benefit of tVNS and GCS to decrease SAS in a driving task when both techniques were applied individually, with better results in the case of tVNS (i.e., less SAS symptomatology with a better body balance leading to better driving performance). Following this study and previous evidence that supports the use of different techniques together to alleviate SAS (e.g., Bos, 2015; Gálvez-García et al., 2020b), the main aim of this study was to test the effectiveness of tVNS and GCS in conjunction at mitigating SAS in a driving task. These two techniques were administered concurrently and individually in active and sham conditions and evaluated in contrast to a control situation where no stimulation was given.

The data pattern confirms that active GCS + tVNS condition decreased SAS more effectively when both techniques were administrated conjointly (93%) than in isolation (69% for active GCS and 83% for active tVNS). In this context, the importance of SSQ scores should be carefully weighed. Stanney et al. (1997) stated that SSQ scores may be linked with negligible (<5), minimal (5–10), significant (10–15), and concerning (15–20) symptoms. Any value >20 means a critical symptomatic factor. This means that participants in the no stimulation condition (i.e., baseline) show high SAS symptomatology (67.05 SSQ scores). However, this is not surprising since previous research where the same driving task was used also showed high SSQ scores in baseline conditions (e.g., Espinoza-Palavicino et al., 2023; Gálvez-García et al., 2020b). This is due to a demanding driving task because participants had to maintain high speeds in the curves, which would facilitate SAS symptoms. As previously stated, this manipulation was fundamental to homogenizing driving variables among participants (since varying speeds, particularly on curves, could interfere with the sickness ratings). In contrast, the isolated administration of active GCS and tVNS reflect SSQ total scores of 21 and 12, respectively. This reflects that although active GCS is a good technique for reducing SAS, it still states severe symptoms. However, active tVNS applied in isolation reflect scores below 15, which means significant symptoms, although not as severe as in active GCS. Importantly, this data pattern also provides further evidence for the previous results of Espinoza-Palavicino et al. (2023) about the remarkable effectiveness of active tVNS in isolation. Thus, our results confirm the administration of tVNS as an effective method to reduce SAS with better results than GCS.

More importantly, active GCS + tVNS condition result in SSQ scores of 4.27. This reflects negligible SAS symptoms, showing a better SSQ reduction than active tVNS and GCS applied in isolation. This confirms our central hypothesis about a better reduction in SAS symptoms when GCS and tVNS were applied concurrently. Moreover, using two techniques to decrease SAS confirms previous literature where different techniques were combined (e.g., Bos, 2015; Gálvez-García et al., 2020a). In this sense, it is essential to highlight that applying active GCS and tVNS together has obtained a 93% reduction of SAS symptoms compared to baseline, which shows greater effectiveness than previous research. As previously stated in the Introduction, the combination of GCS and auditory stimulation reached a 73% reduction in symptoms (Gálvez-García et al., 2015). In a similar vein, the combination of GCS and tactile stimulation showed a 78% reduction (Gálvez-García et al., 2020a). However, although effective, the reported reduction in SAS symptoms was still significant (i.e., scoring above 10 on SSQ total scores). Thus, it can be suggested that the additive use of active GCS and tVNS with negligible symptoms (i.e., scoring under 5 on SSQ total scores) in comparison to GCS and auditory stimulation (Gálvez-García et al., 2015) or GCS and tactile stimulation (Gálvez-García et al., 2020b). Finally, it should be noted that previous research shows large individual differences in susceptibility to simulator sickness. Therefore, SSQ scores often reported larger variations than our results (e.g., Keshavarz et al., 2018). The small variation in SSQ scores in the present study might have been because participants with scores above 65 (75th percentile) or 0 on the Motion Sickness Susceptibility Questionnaire were excluded from the study due to their heightened susceptibility or disinclination to experiencing SAS in the same order. This may have homogenized the differences in susceptibility to simulator sickness. In addition, it may explain the small variation in SSQ scores in previous research with the same inclusion criteria (e.g., Espinoza-Palavicino et al., 2023).

Although we did not address SAS theories in this research, some considerations must be highlighted. Improvements in SAS symptoms due to active conditions in GCS and tVNS are linked with enhanced postural control, as indicated by reduced head movement in both the X-and Y-axes. In this way, the improvement of SAS due to an improvement in postural adjustment could support the postural instability theory (Riccio and Stoffregen, 1991). However, the reduction of symptoms in sham conditions of GCS and tVNS was not based on improving balance ability (i.e., no differences in head sway between sham conditions and baseline). This supports previous studies claiming that the impairment of balance ability is not the only mechanism underlying SAS (Bos, 2015; Keshavarz et al., 2015). More importantly, it supports different approaches to the mechanisms and mitigation of SAS, such as the distraction from symptoms awareness (Reason and Brand, 1975), in line with previous research where different distractors are effective in mitigating SAS (e.g., D’Amour et al., 2017; Gálvez-García et al., 2017). In any case, it should be noted that although effective, the reduction of SAS symptoms in sham conditions still reflected severe symptoms, with active conditions (where there was a positive impact on body balance) being much more effective in reducing SAS symptoms. Moreover, our findings reinforce the idea that SAS is a complex phenomenon influencing body balance and other elements like attention.

Some additional considerations regarding the processes by which active tVNS and GCS reduced SAS should be explained. As noted in the Introduction, the critical and main factor behind the enhancement in SAS may be attributed to the improved body balance from tVNS. However, active tVNS and GCS might also have distracted the participants from the symptoms in a similar way to sham conditions. Moreover, as stated in the Introduction, tVNS could improve different attention-related processes, such as concentration and cognitive flexibility, in line with previous research (Borges et al., 2020; Colzato et al., 2018). This could decrease SAS symptoms by focusing the participant on the task to be performed (i.e., driving) and distracting from the symptoms.

All sham and active conditions for GCS and tVNS improved driving performance in the demanding driving task (i.e., speed constraints), as evidenced by faster speeds accompanied by increased steering movements consistent with prior research with tVNS and GCS (e.g., Espinoza-Palavicino et al., 2023; Gálvez-García et al., 2020b). This improvement is reflected in increased speed and more steering movements, where this driving pattern is optimum for the task. Overall, the results in driving performance confirm the central hypothesis of this study; among the tested conditions, active GCS + tVNS delivery is the most effective technique to improve SAS symptoms and, by extension, driving performance, which is fundamental for its recommendation for future interventions to reduce SAS.

The improvement in driving performance variables negatively correlates with SAS symptoms, reflecting that more conservative driving arises from SAS (Helland et al., 2016). Thus, the increase in driving performance variables could be merely interpreted as a reduction in SAS symptoms. However, other cognitive factors positively influenced by tVNS could have an impact on the motor improvement of driving performance, such as motor inhibition (Jongkees et al., 2018), alertness (Raedt et al., 2011), associative memory (Roosevelt et al., 2006), concentration and cognitive flexibility (Borges et al., 2020; Colzato et al., 2018; both also highlighted as mechanisms that decrease SAS in this discussion), and multitasking (Sommer et al., 2023).

Although the results of our research are promising, future research is needed to generalize our results and to overcome some of the limitations of this research. First, active GCS + tVNS has been proven suitable for decreasing SAS in a demanding driving task where participants must maintain high speeds in the curves, leading to increased SAS symptoms. Thus, exploring different demanding driving tasks and speed constraints should be recommended to extend the applicability of our findings. Second, future research with special populations prone to develop SAS (e.g., elderly drivers; Keshavarz et al., 2018) would help to generalize our results and the overall impact of both techniques in SAS. Third, subsequent research could also employ real-time measurements such as skin conductance (Gavgani et al., 2017; Mwange et al., 2022). Fourth, active tVNS and GCS have been tested as suitable countermeasures to alleviate SAS and improve driving performance. However, other studies focused on the efficacy of tVNS in cognitive processing in driving simulators could obtain results biased by the efficacy of tVNS in SAS. Thus, this factor should be considered an explanatory element (or at least to control it). Fifth, it has been corroborated that active tVNS produces less head movement, decreasing SAS and thus leading to an improvement in driving performance variables. However, the corroboration of the mechanism of SAS reduction by tVNS (i.e., the mentioned relationships) should be further explored by examining mediation and causality (i.e., directional effect). For this purpose, we recommend using structural equation modeling with a large sample size. Sixthly, given the reduction in head movements demonstrated by tVNS and GCS, their utilization may alleviate other forms of motion sickness. This is because individuals prone to motion sickness have been observed to exhibit a greater range of head movements, particularly when traveling in vehicles such as ships, aeroplanes, or cars (Iskander et al., 2019). This is exemplified by carsickness, which is caused by varying lateral accelerations (Kuiper et al., 2018). In this regard, it is recommended that further research be conducted to confirm the benefits of tVNS and GCS in addressing carsickness. Especially in the context of autonomous vehicles, where users may experience variations in acceleration accompanied by the performance of different activities that result in greater head movements in various directions relative to vehicle motion, potentially leading to an increased incidence of motion sickness. Finally, we want to stress that future research lines could test combining more than two techniques to alleviate SAS. For example, tVNS and GCS with auditory stimulation (e.g., Gálvez-García, 2015), habituation and gradual exposure to the simulator which are highly effective (e.g., Heutink et al., 2019), and pleasant olfactory stimulation (Keshavarz et al., 2015) among other countermeasures.

In summary, our results support that tVNS and GCS, when applied jointly, are particularly effective in reducing SAS in a fixed-base simulator, improving driving performance variables. We conclude that SAS symptom reduction is due to an improvement in neuromodulation of motor control that impacts body balance and the driving task itself. All this, along with a distraction of the SAS symptoms and a possible enhancement in other cognitive mechanisms such as concentration, cognitive flexibility, and multitasking. Overall, an effective ergonomic tool for minimizing SAS is proposed. Therefore, we recommend using tVNS and GCS in conjunction to alleviate SAS in future interventions.

## Data Availability

The raw data supporting the conclusions of this article will be made available by the authors, without undue reservation.

## Footnotes

^1^ https://www.rijschool-simulator.nl/
